# Inhibitory Effects of Myricetin on Lipopolysaccharide-Induced Neuroinflammation

**DOI:** 10.3390/brainsci10010032

**Published:** 2020-01-06

**Authors:** Jung-Hee Jang, Seung Hoon Lee, Kyungsook Jung, Horyong Yoo, Gunhyuk Park

**Affiliations:** 1 Department of Neurologic Disorders & Aging Brain Constitution, Dunsan Korean Medicine Hospital, Daejeon 34054, Korea; jee3838@daum.net; 2 Clinical Medicine Division, Korea Institute of Oriental Medicine, Daejeon 34054, Korea; 3 Herbal Medicine Research Division, Korea Institute of Oriental Medicine, Daejeon 34054, Korea; uranos5@kiom.re.kr; 4 Natural Product Material Research Center, Korea Research Institute of Bioscience and Biotechnology, Jeonbuk 56212, Korea; jungks@kribb.re.kr; 5 Herbal Medicine Resources Research Center, Korea Institute of Oriental Medicine, Jeollanam-do 58245, Korea

**Keywords:** myricetin, inflammation, microglia, lipopolysaccharide-induced neuroinflammation, cytokines

## Abstract

Microglial activation elicits an immune response by producing proinflammatory modulators and cytokines that cause neurodegeneration. Therefore, a plausible strategy to prevent neurodegeneration is to inhibit neuroinflammation caused by microglial activation. Myricetin, a natural flavanol, induces neuroprotective effects by inhibiting inflammation and oxidative stress. However, whether myricetin inhibits lipopolysaccharide (LPS)-induced neuroinflammation in hippocampus and cortex regions is not known. To test this, we examined the effects of myricetin on LPS-induced neuroinflammation in a microglial BV2 cell line. We found that myricetin significantly downregulated several markers of the neuroinflammatory response in LPS-induced activated microglia, including inducible nitric oxide (NO) synthase (iNOS), cyclooxygenase-2 (COX-2), and proinflammatory modulators and cytokines such as prostaglandin E2 (PGE_2_), interleukin-1β (IL-1β), and tumor necrosis factor-α (TNF-α). Moreover, myricetin suppressed the expression of c-Jun NH2-terminal kinase (JNK), p38 MAPK, and extracellular signal-regulated kinase (ERK), which are components of the mitogen-activated protein kinase (MAPK) signaling pathway. Furthermore, myricetin inhibited LPS-induced macrophages and microglial activation in the hippocampus and cortex of mice. Based on our results, we suggest that myricetin inhibits neuroinflammation in BV2 microglia by inhibiting the MAPK signaling pathway and the production of proinflammatory modulators and cytokines. Therefore, this could potentially be used for the treatment of neuroinflammatory diseases.

## 1. Introduction

Microglia are found in the central nervous system (CNS) where they play a pivotal role in the immune response and maintaining homeostasis in the brain, protecting the CNS against diverse types of pathogens. Infection, injury and irritants such as lipopolysaccharide (LPS) lead to the activation of microglia and the release of various cytokine and chemokine factors including cyclooxygenase-2 (COX-2) and inducible nitric oxide (NO) synthase (iNOS), inflammatory modulators such as prostaglandin E_2_ (PGE_2_) and NO, proinflammatory cytokines such as interleukin-1β (IL-1β) and tumor necrosis factor-α (TNF-α) [1,2,3,4]. These neuroinflammatory molecules secreted by activated microglia are reported to be associated with mitogen-activated protein kinase (MAPK), nuclear factor (NF)-κB signaling [2,5]. The constant activation of microglia leads to excessive production of cytotoxic mediators and neuroinflammation that contributes to neurodegeneration. It is closely related to neurodegenerative diseases such as Alzheimer’s disease (AD), Parkinson’s disease (PD), Huntington’s disease, and multiple sclerosis [6,7,8,9]. Therefore, inhibiting neuroinflammatory events in microglia may be a promising strategy for preventing the progression of neuroinflammatory-modulated neurodegenerative diseases.

**Figure 1:**
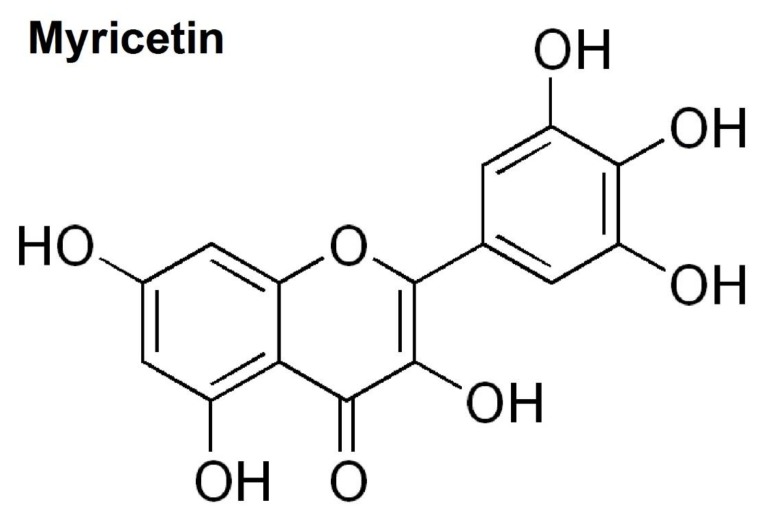
Chemical structure of myricetin.

Myricetin (3,5,7,3′,4′,5′-hexahydroxyflavone cannabiscetin) is a natural flavanol extracted from vegetables, tea, fruits, berries, red wine, and medical plants. According to a recent study, myricetin is believed to be present in *Euphorbia dracunculoides*, which has therapeutic effects for diseases such as rheumatism, epilepsy, edema, and so on [10]. Myricetin is structurally similar to quercetin, morin, kaempferol, and fisetin, and is reported to have many similar functions as these other members of the flavanol class of flavonoids. The chemical structure of myricetin is shown in Figure 1.

Myricetin reportedly has antioxidant, anticarcinogen, antiviral, anti-inflammation, antidiabetic, and antiatherosclerotic pharmacological actions [11]. Recently, the neuroprotective effects of myricetin were demonstrated [12]. Myricetin was reported to attenuate neuronal injury by anti-oxidation and anti-inflammation [12]. In a rat middle cerebral artery occlusion model, myricetin reduced ischemic cerebral injury associated with the MAPK, NF-κB/p65, and protein kinase B (AKT) signaling pathways [12]. Furthermore, in mice subjected to restraint stress to induce cognitive deficits and depression, myricetin improved spatial memory and depression-like behavior. Myricetin also normalized the decreased brain-derived neurotrophic factor (BDNF) levels observed in the hippocampus [13,14]. Myricetin was also found to protect 1-methyl-4-phenylpyridinium (MPP+)-treated MES23.5 dopaminergic cells—which exhibit similar properties to primary neurons originating in the substantia nigra, a lesion of PD—by inhibiting MAPK kinase and c-Jun N-terminal kinase (JNK) activation and having an anti-oxidative role [15]. In addition, myricetin was found to have neuroprotective effects in 6-hydroxydopamine-induced dopamine degeneration [16] and a rotenone-induced Drosophila model of PD [17]. Taken together, these results point to the neuroprotective, anti-oxidative, and anti-inflammatory effects of myricetin; however, whether myricetin has similar effects on LPS-mediated neuroinflammation in the hippocampus and cortex has not yet been investigated.

In the present study, we investigated the anti-inflammatory effects of myricetin in LPS-activated macrophages and microglial activation in the hippocampus and cortex. In addition, we investigated the potential mechanisms underlying the pharmacological effects of myricetin by assessing the response of the MAPK and NF-κB signaling pathway and inflammatory modulators. We found that myricetin inhibited LPS-induced inflammatory effects by inhibiting the MAPK signaling pathway.

## 2. Materials and Methods 

### 2.1. Chemicals and Reagents

Dulbecco’s modified Eagle’s medium (DMEM), penicillin-streptomycin, and fetal bovine serum (FBS) were purchased from Gibco (Gaithersburg, MD, USA). Phosphate buffered saline (PBS), 3-(4,5-dimethylthiazol-2-yl)-2,5-diphenyl tetrazolium bromide (MTT), dimethylsulfoxide (DMSO), LPS, nitric oxide and myricetin were purchased from Sigma-Aldrich (St. Louis, MO, USA). Rabbit PGE_2_ and phosphoTracer ERK1/2 (pT202/Y204), p38 MAPK (pT180/Y182), and JNK1/2/3 (pT183/Y185) ELISA kits were purchased from Abcam (ab119674, Cambridge, UK). TNF-α and IL-1β were obtained from R&D systems (Minneapolis, MN, USA). iNOS and COX-2 were obtained from cell signaling (Beverly, MA, USA). Rabbit anti-Ionized calcium binding adaptor molecule 1 (IBA-1) was obtained from Wako Pure (019-19741, Tokyo, Japan). Biotinylated goat anti-rabbit antibody and avidin-biotin complex (ABC) were purchased from Vector Laboratories, Inc. (Burlingame, CA, USA). All other reagents used were of guaranteed or analytical grade.

### 2.2. Cell Culture and Cytotoxicity Measurements

Cells were maintained and cytotoxicity was performed as described previously [18,19]. Briefly, BV2 cells were treated with myricetin at concentrations of 0.1–50 µM for 1 h, subsequently stimulated with LPS for an additional 23 h, and incubated with 1 mg/mL MTT for 2 h. 

### 2.3. Measurement of Nitric Oxide (NO), MAPK Signaling, TNF-α, IL-1β, PGE_2_, iNOS, and COX-2 Levels

The NO synthesis was analyzed by determining the accumulation of nitrite (NO2-) in culture supernatant using the Griess Reagent System (Promega, Madison, WI, USA). The supernatant and sulphanilamide solution were mixed for 10 min at 25–28 °C, then added to a N-(1-Naphthyl)ethylenediamine solution for an additional 5 min. The absorbance was measured at 540 nm by using a spectrophotometer (Versamax microplate reader, Molecular Device). MAPK signaling, TNF-α (MTA00B), IL-1β (MLB00C), PGE2 (ab133021), iNOS (#7097), and COX-2 (#7291) were measured using an ELISA kit according to the manufacturer’s instructions.

### 2.4. Animals

Male mice (8 weeks, 23–24 g) were purchased from Orient-Bio Inc. (Seoul, Korea) and maintained under temperature- and light-controlled conditions (20–23 °C, 12 h light/12-h dark cycle) with food and water provided ad libitum. All animals were acclimatized for 7 days prior to drug administration. The experimental protocol was approved by the institutional animal care committee of KRIBB (KRIBB-AEC-19185) and performed according to the guidelines of the Animal Care and Use Committee at the Korea Research Institute of Bioscience and Biotechnology.

### 2.5. Drug Administration

Mice were assigned to 1 of 4 groups: (1) control; (2) LPS; (3) LPS + myricetin 50 mg/kg/day; and (4) LPS+ myricetin 100 mg/kg/day. Myricetin (dissolved in saline) was administered intraperitoneally once per day for 7 days. 2 h after the last drug administration, LPS (dissolved in saline) was injected intraperitoneally at a dose of 5 mg/kg [18,19].

### 2.6. Tissue Preparation and Immunohistochemistry

At 2 h after LPS injection, after transcardiac perfusion, brains were rapidly taken out, post-fixed in 4% paraformaldehyde (PFA) overnight, then cryoprotected in 30% sucrose. Free floating sections were rinsed in PBS at 25–28 °C before immunostaining, and pre-treated with 1 % H_2_O_2_ to block endogenous peroxidase activity. Next, they were incubated overnight with rabbit anti-IBA-1 antibody (1:500 dilutions). The sections were incubated with a biotinylated anti-rabbit IgG (1:200 dilutions) and then with the ABC solution for 1 h. The color was developed with 3,3′-diaminobenzidine. Finally, the sections were mounted on gelatin-coated slides, dried, dehydrated in an ascending alcohol, and cleared in xylene.

### 2.7. Statistical Analysis

The statistics are expressed as the mean ± standard error of the mean (SEM). The statistical variables were analyzed using a one-way analysis of variance (ANOVA) and post-hoc multiple mean comparisons (Tukey’s HSD test). The statistical significance was set at *p* < 0.05. All variables were analyzed using the GraphPad Prism 5.10 software (GraphPad Software Inc., San Diego, CA, USA).

## 3. Results

### 3.1. Effects of Myricetin on LPS-Induced Cytotoxicity and NO Generation in Microglia BV2 Cells

**Figure 2:**
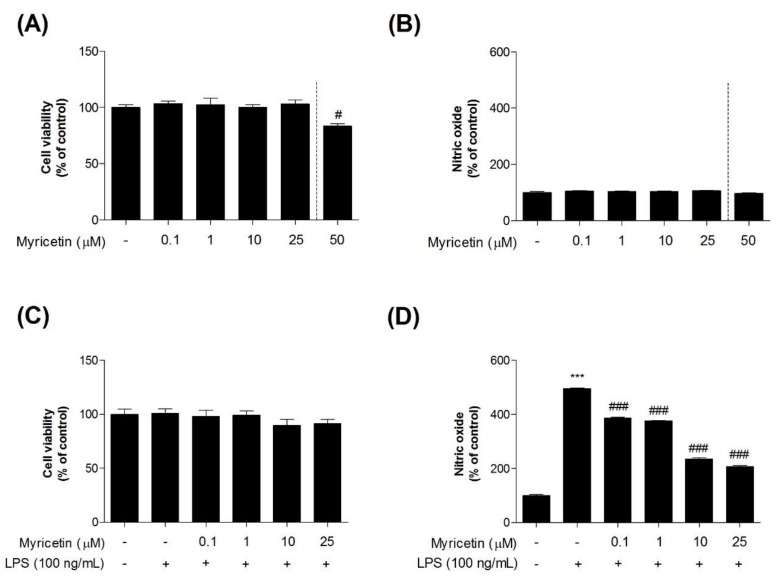
The effect of myricetin on LPS-induced NO production in microglia BV2 cells. Cells were treated with myricetin for 1 h, and then stimulated with LPS for an additional 23 h. (**a**,**c**) The cell viability was assessed using an MTT assay. (**b**,**d**) The culture supernatant was also subjected to nitrite quantification. Values are means ± standard error of the mean. *** *p* < 0.001 compared to the control group, and # *p* < 0.05 and ### *p* < 0.001 compared to the LPS-alone group. LPS, lipopolysaccharide; NO, nitric oxide.

To investigate the inhibitory effects of myricetin on LPS-induced toxicity in microglia BV2 cells, we measured cytotoxicity using an MTT assay. Treatment with myricetin at 0.1–25 μM alone had no effect on the cells (Figure 2a–c). However, myricetin treatment at 50 μM caused increased cytotoxicity (Figure 2a). Therefore, the experiments described below were performed at a myricetin treatment dose of 0.1–25 μM. While cells exposed to LPS showed significant NO generation compared to control cells (232.20 ± 7.84%), pre-treatment with myricetin at 0.1–25 μM (232.20 ± 7.84 - 232.20 ± 7.84%) inhibited this effect (Figure 2d).

### 3.2. Inhibitory Effects of Myricetin on LPS-Induced iNOS and COX-2 Levels

**Figure 3:**
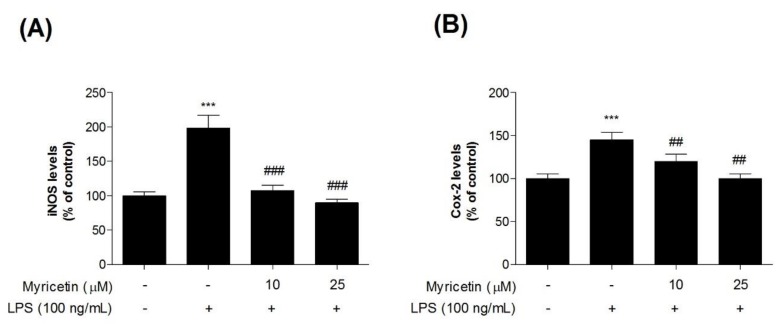
The effect of myricetin on LPS-induced iNOS and COX-2 levels in microglia BV2 cells. Cells were treated with myricetin for 1 h, and then stimulated with LPS for an additional 23 h. (**a**) iNOS and (**b**) COX-2 levels were measured by ELISA kit. Values are means ± standard error of the mean. *** *p* < 0.001 compared to the control group, and ## *p* < 0.01 and ### *p* < 0.001 compared to the LPS-alone group. LPS, lipopolysaccharide; iNOS, inducible nitric oxide synthase; COX-2, cyclooxygenase-2.

To evaluate the inhibitory effects of myricetin on LPS-induced inflammatory mediators in microglia BV2 cells, we measured iNOS and COX-2 levels using ELISA kits. Treatment with LPS significantly increased iNOS and COX-2 levels compared with the control cells (by 198.52 ± 18.44% and 145.41 ± 8.58, respectively), while treatment with 10 or 25 μM myricetin decreased LPS-induced iNOS and COX-2 (by 107.70 ± 7.41 and 89.75 ± 5.04%, and 119.99 ± 8.26, and 100.69 ± 5.63%, respectively) (Figure 3a,b).

### 3.3. Inhibitory Effects of Myricetin on LPS-Induced TNF-α, IL-1β, and PGE_2_ Levels

**Figure 4:**
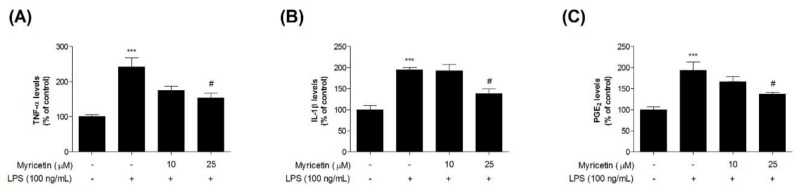
The effect of myricetin on LPS-induced TNF-α, IL-1β, and PGE_2_ levels in microglia BV2 cells. Cells were treated with myricetin for 1 h, and then stimulated with LPS for an additional 23 h. (**a**) TNF-α, (**b**) IL-1β, and (**c**) PGE_2_ levels were measured by ELISA kits. Values are means ± standard error of the mean. *** *p* < 0.001 compared to the control group, and # *p* < 0.05 compared to the LPS-alone group. LPS, lipopolysaccharide; TNF-α, tumor necrosis factor-α; IL-1β, interleukin-1β; PGE2, prostaglandin E2.

To evaluate the inhibitory effects of myricetin on LPS-induced inflammatory cytokines in microglia BV2 cells, we measured TNF-α, IL-1β, and PGE_2_ using ELISA kits. Treatment with LPS significantly increased TNF-α, IL-1β, and PGE_2_ levels compared with the control cells (by 241.83 ± 26.07, 194.75 ± 5.40, and 193.71 ± 19.25%, respectively), while treatment with 10 or 25 μM myricetin decreased LPS-induced TNF-α, IL-1β, and PGE_2_ levels (by 174.51 ± 12.69–153.68 ± 13.76, 192.87 ± 14.30–137.94 ± 11.36, and 193.71 ± 12.06–136.78 ± 5.21%, respectively) (Figure 4a,b).

### 3.4. Inhibitory Effects of Myricetin on LPS-Induced Phosphor-MAPKs Signaling ERK, JNK, and p38 Levels

**Figure 5:**
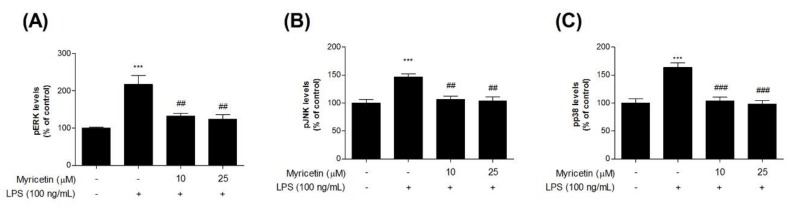
The effect of myricetin on LPS-induced MAPK signaling by measuring p-ERK, p-JNK, and p-p38 levels in microglia BV2 cells. Cells were treated with myricetin for 1 h, and then stimulated with LPS for an additional 30 min or 23 h. (**a**) MAPK signaling p-ERK, (**b**) p-JNK, and (**c**) p-p38 levels were measured by ELISA kit. Values are means ± standard error of the mean. *** *p* < 0.001 compared to the control group, and ## *p* < 0.01 and ### *p* < 0.001 compared to the LPS-alone group. LPS, lipopolysaccharide; MAPK, mitogen-activated protein kinase; ERK, extracellular signal-regulated kinase; JNK, c-Jun NH2-terminal kinase, p38, p38 MAPK.

To evaluate the inhibitory effects of myricetin on LPS-induced MAPK signaling in microglia BV2 cells, we measured phosphorylated (p-) MAPKs (ERK, JNK, and p38) using ELISA kits. Treatment with LPS significantly increased p-ERK, p-JNK, and p-p38 levels compared with the control cells (by 217.45 ± 24.44, 147.02 ± 5.01, and 163.95 ± 8.05%, respectively), while treatment with 10 or 25 μM myricetin decreased LPS-induced p-MAPK ERK, JNK, and p38 levels (by 132.11 ± 8.20-124.62 ± 11.32, 105.84 ± 6.57-103.82 ± 7.29, and 103.94 ± 6.45-98.21 ± 5.97%, respectively) (Figure 5a–c).

### 3.5. Inhibitory Effects of Myricetin on LPS-Induced Activation of Microglia in Hippocampus and Cortex in Mice

**Figure 6:**
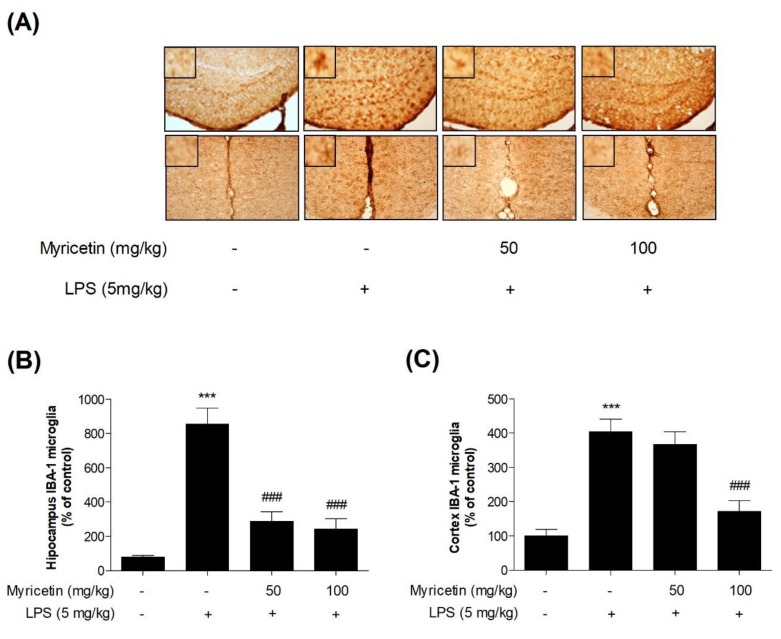
The effect of myricetin on LPS-induced microglial activation in the hippocampus and cortex in mice. Following myricetin administration and LPS treatment in mice, microglia activation was measured by IHC. (**a**,**b**) The number of activated microglia in the hippocampus and (**a**,**c**) cortex were measured. Scale bars = 250 µm. Values are presented as means ± standard error of the mean. * *p* < 0.05 and ** *p* < 0.01 compared with the control group, #*p* < 0.05, ## *p* < 0.01, and ### *p* < 0.001 compared with the LPS-treated group. LPS, lipopolysaccharide; IHC, immunohistochemistry.

To evaluate the anti-inflammatory effect of myricetin in vivo, neuroinflammation was induced by LPS. IBA-1 is a microglia/macrophage-specific calcium-binding protein whose levels increase in activated microglia. As expected, the levels of IBA-1 were significantly increased (by 1069.48 ± 139.06% and 403.91 ± 33.29%, respectively) in the hippocampus and cortex of the LPS-treated group. Myricetin suppressed this decrease of microglial activation (by 360.71 ± 82.63% - 259.09 ± 93.68% and 367.45 ± 32.83% - 172.35 ± 27.65%, respectively) (Figure 6a–c). These results suggest that myricetin regulates neuroinflammation by suppressing microglial activation in the hippocampus and cortex of the mouse brain.

## 4. Discussion

Microglia contribute to the development of the CNS, the maintenance and plasticity of neuronal networks, and the immune response. However, excessive activation of microglia produces proinflammatory cytokines and neurotoxic materials that lead to neurodegeneration [2,9]. Therefore, inhibiting neuroinflammation by modulating the microglial response may help prevent neurodegenerative diseases. This is the first study to investigate inhibitory effects of myricetin on LPS-induced neuroinflammation in a microglia BV2 cell line. In the present study, we found that myricetin prevented LPS-induced neuroinflammation by suppressing proinflammatory cytokines (IL-1β and TNF- α), the overexpression of NO and PGE_2_, and protein overexpression of iNOS and COX-2, by interfering with MAPK signaling pathways.

Many studies have reported that COX-2 and iNOS are induced in various CNS diseases [20,21,22,23]. NO, derived from iNOS, is a major neuroinflammatory modulator and excessive NO production occurs in both acute and chronic neuroinflammation [20,23]. High levels of NO induce COX-2 expression. Similarly, a well-known neuroinflammatory modulator, PGE_2_, which is produced by COX-2 from arachidonic acid, contributes to the development of many chronic neuroinflammatory diseases [24]. Therefore, blocking the production of these modulators has been a target for therapeutic anti-inflammatory drugs. These findings demonstrated that treatment of LPS stimulated BV2 microglia with myricetin significantly inhibited the generation of NO. In combination with inhibitory effects on NO, we also found that iNOS secretion was suppressed by myricetin treatment at 12 and 25 μM. Additionally, pretreatment with myricetin attenuated the secretion of COX-2 in LPS stimulated BV2 microglia. These results suggest that a significant decrease in NO release by myricetin is associated with suppression of iNOS. Therefore, any substance that can attenuate the secretion of iNOS and COX-2 may be useful for preventing the progression of neurodegeneration disease. Various proinflammatory cytokines also play major roles in neuroinflammation [2]. Naturally derived compounds that inhibit the generation of proinflammatory cytokines could be alternative anti-inflammatory agents. Inhibitory effects of phytochemicals on the generation of proinflammatory cytokines have been extensively studied to develop anti-inflammatory agents to inhibit inflammatory diseases [2,4,25]. In this study, we demonstrated that myricetin remarkably inhibited the generation of TNF-α, IL-1β and PGE_2_ in LPS-stimulated BV2 microglia. Moreover, myricetin reduced the activation of microglia in LPS-treated hippocampus and cortex regions of mice. Thus, the present findings further support the potential of myricetin as a neuroprotective agent by inhibiting inflammation.

MAPKs modulate various cellular activities including cyto-proliferation, differentiation, apoptosis, inflammation, and innate immunity [26,27,28]. MAPKs are intracellular serine/threonine protein kinases consisting of JNK, p38 MAPK, and ERK [26,29]. The molecular mechanisms of MAPK activation have been well studied and involve the activation of a signaling cascade for cytoplasmic and nuclear translocation, and activation of transcription factor 2. MAPK signaling pathways are activated and triggered by cellular stress, microbial components such as bacterial LPS, and proinflammatory cytokines such as PGE_2,_ IL-1β, TNF-α [26,28,29]. In recent studies, the attenuation of inflammation and oxidative stress by myricetin was associated with the MAPK signaling pathway. It was shown that myricetin reduced the phosphorylation of p38 MAPK in ischemia induced cerebral injury [12] and MAPK4 and JNK in MPP^+^ treated MES23.5 cells [15]. Myricetin also attenuated LPS-induced inflammation in RAW 264.7 macrophages and lung injury mouse models by suppressing JNK, p-ERK and p38 in MAPK signaling pathway [30]. Furthermore, myricetin exhibited a cytoprotective effect against H_2_O_2_-induced apoptosis in Chinese hamster lung fibroblast. Myricetin increased the level of anti-apoptotic factor (BCL-2) and decreased the levels of pro-apoptotic factors (BAX, CASPASE-9 and -3). It was suggested that myricetin prevents oxidative stress-induced apoptosis via the MAPK signaling pathway [31]. On the basis of these findings, the inhibitory effects of myricetin on LPS-induced MAPK activation was investigated in this study. Here, the anti-oxidative and inflammatory effects of myricetin was confirmed via MAPK signaling. We found that myricetin treatment significantly suppressed LPS-induced phosphorylation of ERK, JNK, and p38. These findings suggest that myricetin inhibits the production of inflammatory modulators by inhibiting the MAPK signaling pathway. 

Neurodegenerative diseases are characterized by neuronal degeneration in specific regions of the CNS [32]. More specifically, the hippocampus is known to be severely damaged during AD in the brain [33]. Neurodegeneration is mediated by inflammatory mediators and neuroinflammation by activated microglia, astrocytes, and neurons. Therefore, we investigated the effects of myricetin on microglial activation and observed that microglial activation by LPS in the hippocampus and cortex of the mouse brain was significantly suppressed following myricetin treatment. A previous study on the neuroprotective effects of myricetin in specific regions of the CNS showed that myricetin markedly increased the number of hippocampal neurons in rats with AD [34]. Similarly, myricetin was reported to have a neuroprotective effect by inhibiting excessive glutamate release, which is known to kill neurons and lead to neurological disorders by decreasing K^+^ channel blocker 4-aminopyridine and voltage-dependent Ca^2+^ entry in rat cerebrocortical nerve terminals [35]. In addition, it was demonstrated that myricetin is a lipophilic compound and can pass through biological membranes such as the blood-brain barrier [36]. Based on these finding, we hypothesize that myricetin may play a role in improving chronic brain disorder by crossing the blood-brain barrier. However, to date, there have only been a few studies confirming the effect of myricetin on brain diseases. To confirm the therapeutic potential of myricetin for neurodegenerative disease, further study is need such as investigating the effects of myricetin on immune cells in several animal models of neurodegenerative disease, and the changes in activated microglial morphology and macrophage-like capabilities such as amoeboid cell shape, in brain tissue by myricetin. In particular, further study of the specific active inhibition of microglia is required, since the responses of microglia and macrophage were not studied separately as the limits of this study.

## 5. Conclusions

We found that myricetin treatment of LPS-induced BV2 microglia significantly inhibited the secretion of iNOS and COX-2, as well as attenuated the release of LPS-stimulated proinflammatory modulators and cytokines. These inhibitory effects were related to the attenuation of inflammatory-modulated MAPK signaling pathway activation. We also found that myricetin inhibited LPS-induced activation of macrophages and microglia in the hippocampus and cortex region. Therefore, the attenuation of neuroinflammation by myricetin, as demonstrated in this study, could be advantageous in the treatment of neurodegenerative diseases.
